# Characterization of Domiphen Bromide as a New Fast-Acting Antiplasmodial Agent Inhibiting the Apicoplastidic Methyl Erythritol Phosphate Pathway

**DOI:** 10.3390/pharmaceutics14071320

**Published:** 2022-06-22

**Authors:** Arnau Biosca, Miriam Ramírez, Alex Gomez-Gomez, Aritz Lafuente, Valentín Iglesias, Oscar J. Pozo, Santiago Imperial, Xavier Fernàndez-Busquets

**Affiliations:** 1 Barcelona Institute for Global Health (ISGlobal, Hospital Clínic-Universitat de Barcelona), Rosselló 149-153, 08036 Barcelona, Spain; naubiosca@gmail.com (A.B.); miriam.ramirez@isglobal.org (M.R.); aritz.lafuente@gmail.com (A.L.); valentin.iglesias.mas@gmail.com (V.I.); 2 Nanomalaria Group, Institute for Bioengineering of Catalonia (IBEC), The Barcelona Institute of Science and Technology, Baldiri Reixac 10-12, 08028 Barcelona, Spain; 3 Nanoscience and Nanotechnology Institute (IN2UB), University of Barcelona, Martí i Franquès 1, 08028 Barcelona, Spain; simperial@ub.edu; 4 Integrative Pharmacology and Systems Neuroscience Research Group, Neurosciences Research Program, IMIM-Institut Hospital del Mar d’Investigacions Mèdiques, Doctor Aiguader 88, 08003 Barcelona, Spain; agomez@imim.es (A.G.-G.); opozo@imim.es (O.J.P.); 5 Department of Experimental and Health Sciences, Universitat Pompeu Fabra, Doctor Aiguader 88, 08003 Barcelona, Spain; 6 Institut de Biotecnologia i Biomedicina and Departament de Bioquímica i Biologia Molecular, Universitat Autònoma de Barcelona, 08193 Bellaterra, Spain; 7 Department of Biochemistry and Molecular Biomedicine, University of Barcelona, Avda. Diagonal 643, 08028 Barcelona, Spain

**Keywords:** malaria, *Plasmodium falciparum*, domiphen bromide, methyl erythritol phosphate pathway, antimalarial drugs, antibiotics

## Abstract

The evolution of resistance by the malaria parasite to artemisinin, the key component of the combination therapy strategies that are at the core of current antimalarial treatments, calls for the urgent identification of new fast-acting antimalarials. The apicoplast organelle is a preferred target of antimalarial drugs because it contains biochemical processes absent from the human host. Fosmidomycin is the only drug in clinical trials targeting the apicoplast, where it inhibits the methyl erythritol phosphate (MEP) pathway. Here, we characterized the antiplasmodial activity of domiphen bromide (DB), another MEP pathway inhibitor with a rapid mode of action that arrests the in vitro growth of *Plasmodium falciparum* at the early trophozoite stage. Metabolomic analysis of the MEP pathway and Krebs cycle intermediates in 20 µM DB-treated parasites suggested a rapid activation of glycolysis with a concomitant decrease in mitochondrial activity, consistent with a rapid killing of the pathogen. These results present DB as a model compound for the development of new, potentially interesting drugs for future antimalarial combination therapies.

## 1. Introduction

In 2006 the World Health Organization (WHO) recommended artemisinin-based combination therapies as global first-line treatments for *Plasmodium falciparum* malaria [1]. This therapeutic strategy consists of the combination of a fast-acting antimalarial (artemisinin) with a second drug with a longer blood residence time and, ideally, a different mode of action, which wipes out those parasites that were not killed by the first compound. However, by 2016, the emergence of artemisinin and partner-drug resistance in *P. falciparum* was detected in the Greater Mekong Subregion [2], and recently the independent evolution of artemisinin resistance has also been reported in Africa [3] and South America [4]. This alarming scenario calls for the urgent development of new fast-acting drugs with little-exploited targets in the malaria parasite.

Isoprenoids are a large and diverse class of naturally occurring organic chemicals which are essential for cell survival in all organisms [5]. All isoprenoids are derived from the universal five-carbon precursors isopentenyl diphosphate (IPP) and dimethylallyl diphosphate (DMAPP), which may be synthesized via one of two independent nonhomologous pathways: the classical mevalonate pathway or the alternative 2-*C*-methyl-D-erythritol 4-phosphate/1-deoxy-D-xylulose 5-phosphate (MEP/DXP) pathway [6]. In the malaria parasite, this biosynthetic route takes place in the apicoplast, a relict, plastid-like organelle [7,8] that gives its name to the Apicomplexa phylum, which also includes important human and animal pathogens such as *Toxoplasma, Babesia, Cryptosporidium*, *Cyclospora*, and *Cystoisospora*. Malaria parasites lacking the apicoplast are entirely dependent on exogenous IPP for survival, indicating that isoprenoid precursor biosynthesis is the only essential function of the apicoplast during blood stage growth [9].

**Figure 1:**
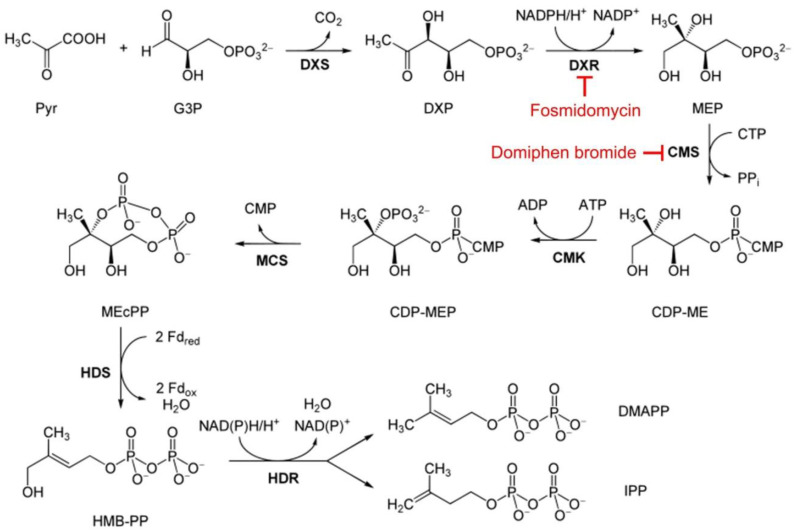
MEP pathway in *P. falciparum*. Abbreviations: pyruvate (Pyr), glyceraldehyde 3-phosphate (G3P), 1-deoxy-D-xylulose 5-phosphate (DXP), DXP synthase (DXS), DXP reductoisomerase (DXR), 2-*C*-methyl-D-erythritol 4-phosphate (MEP), MEP cytidylyltransferase (CMS), 4-diphosphocytidyl-2-*C*-methylerythritol (CDP-ME), CDP-ME kinase (CMK), CDP-ME 2-phosphate (CDP-MEP), 2-*C*-methyl-D-erythritol 2,4-cyclodiphosphate (MEcPP), MEcPP synthase (MCS), (*E*)-4-hydroxy-3-methyl-but-2-enyl pyrophosphate (HMB-PP), HMB-PP synthase (HDS), HMB-PP reductase (HDR), dimethylallyl pyrophosphate (DMAPP), isopentenyl pyrophosphate (IPP). Adapted from the scheme with permission from [18] Copyright 2010 John Wiley and Sons.

For most bacteria and Apicomplexa parasites, the MEP pathway is the only route for the biosynthesis of IPP and DMAPP and has therefore been identified as an interesting target for the development of new antibiotics and antiparasitic drugs [10,11,12,13,14,15,16]. The MEP pathway in *Plasmodium* starts with the condensation of pyruvate and glyceraldehyde 3-phosphate (G3P), which yields DXP as a key metabolite. DXP reductoisomerase (DXR) then catalyzes the simultaneous intramolecular rearrangement and reduction of DXP to form MEP. The third enzyme of the MEP pathway, 2-*C*-methyl-D-erythritol-4-phosphate cytidylyltransferase (CMS), catalyzes the formation of 4-diphosphocytidyl-2-*C*-methyl-D-erythritol (CDP-ME) [17]. Several reaction steps are necessary for the final conversion of CDP-ME to IPP and DMAPP (Figure 1).

**Figure 2:**
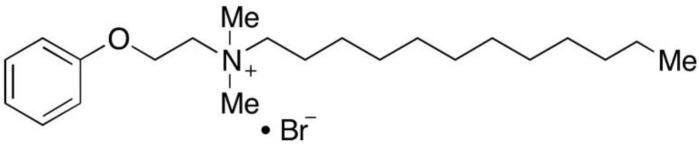
Chemical structure of DB.

One of the most studied MEP pathway inhibitors is fosmidomycin, a compound that blocks DXR and CMS [19], and which has antiplasmodial activity with an in vitro IC50 between 0.3 and 1.2 μM in parasite cultures [10,20]. Exogenous supplementation of the culture with IPP rescued asexual parasites treated with 100 μM fosmidomycin [9], which illustrated the specificity of the drug. Similarly, apicoplast-lacking parasites could only differentiate into gametocytes when the culture was supplemented with IPP [21], thus also indicating the essential role of this metabolite for sexual development and suggesting that other MEP inhibitors could have transmission-blocking activity. Fosmidomycin, which had been proposed as a partner drug in antimalarial combination therapies [22], is now under phase II clinical trials in combination with piperaquine [23]. Other compounds currently under study are fluoropyruvate, which targets the first enzyme of the pathway [24], and MMV008138, obtained from the Malaria Box [25], which inhibits CMS [17]. Similarly, the quaternary ammonium compound domiphen bromide (DB, Figure 2) has been shown to inhibit in vitro CMS from *Mycobacterium smegmatis* [26] and from *Plasmodium vivax* [27], suggesting that it could become a novel antimalarial drug. Recently, DB has also been found to be active against *P. falciparum* cultures, with an IC50 around 1 µM [28]. This compound is soluble in water at 1 g/mL [29], it has a lowest published lethal dose of 10 mg/kg [30,31], and it exhibits antibiotic activity against several bacteria [32,33].

Given the alarming scarcity of antimalarial drugs, the slow rate at which they are developed, and the quick evolution of resistance by the parasite, we initiated an exploration of the potential of DB for malaria therapeutics. We characterized the effect of DB on in vitro *P. falciparum* cultures in comparison with fosmidomycin, using microscopy to observe the effect of both drugs on parasitized red blood cells, and liquid chromatography–electrospray ionization–tandem mass spectrometry (LC–ESI–MS/MS) to analyze the levels of MEP pathway metabolites along time.

## 2. Materials and Methods

Except where otherwise indicated, reagents were purchased from Merck (Darmstadt, Germany).

### 2.1. P. falciparum Cultures and In Vitro Growth Inhibition Assays

*P. falciparum* 3D7 was grown in vitro in human red blood cells of blood group type B prepared as described elsewhere [34], using previously established conditions [35]. Briefly, parasites (thawed from glycerol stocks) were cultured at 37 °C in T25 flasks (SPL Life Sciences Co., Ltd., Naechon-Myeon, South Korea) in Roswell Park Memorial Institute (RPMI) complete medium (containing 5 g/L Albumax II and supplemented with 2 mM glutamine) under a gas mixture of 92% N_2_, 5.5% CO_2_, and 2.5% O_2_. Synchronized ring stage cultures were obtained by 5% sorbitol lysis [36], and the medium was changed every 2 days maintaining 3% hematocrit. A total of 200 μL of these *Plasmodium* cultures with a 7% parasitemia and synchronized at ring stages were plated in 96-well plates and incubated for 48 h at 37 °C in the presence of 20 µM fosmidomycin and/or 20 µM DB added from 5 mg/mL and 20 mM stocks prepared in H_2_O and methanol, respectively. These concentrations were chosen because they are closer to the curative doses to be administered to mice in future in vivo assays, according to our previous data obtained with chloroquine [37,38]. A sample treated with 20 µM chloroquine was also included as a growth inhibition control. The culture was monitored at different times by microscopy (Olympus IX51, Tokyo, Japan), and parasitemia was determined by microscopic counting of blood smears or by flow cytometry as previously described [39]. Just before drug incorporation, and 30 min, 1 h, 8 h, 12 h and 24 h afterwards, 10 mL of culture were removed, spun down at 500× *g* for 5 min, and the resulting pellets instantly frozen by immersion in liquid N_2_. The frozen pellets were lyophilized and stored at −80 °C until LC–ESI–MS/MS analysis.

### 2.2. Determination of Metabolites from the MEP Pathway and the Tricarboxylic Acid Cycle

The frozen lyophilized pellets were first reconstituted in 100 µL of ultrapure water. For the determination of phosphate metabolites of the MEP pathway, 20 µL of the reconstituted extract was mixed with 10 µL of AMP-^13^C_5_ (Toronto Research Chemicals, North York, ON, Canada) used as internal standard, and 70 µL of high-performance liquid chromatography-grade acetonitrile. The mixture was vigorously shaken and centrifuged (5 min, 13,400× *g*), and the supernatant was transferred to a clean vial. Ten microliters was injected into the LC–ESI–MS/MS system, which consisted of an Acquity ultra-performance liquid chromatography system (Waters Associates, Milford, MA, USA) coupled to a Xevo TQ-S micro-triple-quadrupole mass spectrometer provided with an orthogonal Z-spray–electrospray interface (Waters Associates). Nitrogen was used as both drying gas (1200 L/h) and nebulizing gas (50 L/h). The selected capillary voltage was 3 kV in positive ionization and negative mode. The nitrogen desolvation temperature was 600 °C and the source temperature 150 °C. The collision gas was argon at a flow of 0.21 mL/min. The chromatographic separation was performed at 55 °C using an Acquity UPLC^®^ BEH Amide 1.7 µm (2.1 × 100 mm) column at a flow rate of 400 µL/min. The aqueous mobile phase was composed of ultrapure water with ammonium bicarbonate (4 mM, pH 10.5), and the organic mobile phase was composed of a mixture (8:2) of acetonitrile and water with ammonium bicarbonate (100 mM, pH 10.5). The gradient linearly changed the percentage of aqueous mobile phase as follows: 0 min, 5%; 0.5 min, 5%; 2.75 min, 20%; 3.75 min, 20%; 3.76 min, 5%; 4.75 min, 5%. MS/MS detection was performed by a selected reaction monitoring method including two ion transitions per compound: MEP (215 > 79, 215 > 97); DXP (213 > 79, 213 > 97); G3P (169 > 79, 169 > 97); phosphoenolpyruvate (167 > 79, 167 > 97); AMP-^13^C_5_ (351 > 78.9). The first transition was used for quantification. The analysis batch also included standards of MEP, DXP, G3P, and phosphoenolpyruvate to confirm the detection of the compounds.

The determination of pyruvate and other acidic metabolites, including lactate and acidic compounds from the tricarboxylic acid cycle, was performed as previously described [40,41]. Briefly, 5 μL of the reconstituted extract was mixed with 30 μL of an internal standard consisting of a mixture containing 100 ng/mL for succinate-d_4_ and fumarate-^13^C_4_, 1.2 μg/mL for lactate-^13^C_3_, and 10 μg/mL for malate-d_3_ and citrate-d_4_ in ultrapure water. Derivatization was performed by adding 100 μL of a freshly prepared *O*-benzylhydroxylamine: *N*-(3-dimethylaminopropyl)-*N*′-ethylcarbodiimide–hydrochloride mixture to the sample vial. After 60 min of reaction at room temperature, 1 mL of ultrapure water was added to stop the reaction and the mixture was extracted with 4 mL of ethyl acetate. After centrifugation (2000× *g*, 5 min), the organic layer was separated and dried under a nitrogen stream in a water bath at 40 °C and 15 psi. The extracts were reconstituted in 150 μL of ultrapure water:methanol (1:1). Finally, 10 μL of the mixture was injected into the LC–ESI–MS/MS system. Two transitions were acquired per analyte. Both chromatographic and MS/MS conditions can be found elsewhere [41].

Data were processed with the MassLynx software V4.1 (Waters Associates), using the TargetLynx package for integration and data management.

### 2.3. Statistical Analysis

Statistical analyses were performed using Graphpad Prism v6 software (GraphPad Software Inc., La Jolla, CA, USA). Three replicates were taken for each measure. Statistical differences were assessed with the non-parametrical Mann–Whitney U test. *p* < 0.05 was considered statistically significant. In the graphs, *, **, ***, and **** indicate *p* < 0.05, *p* < 0.005, *p* < 0.0005, and *p* < 0.0001, respectively.

### 2.4. Ethics Statement

The human blood used in this work was commercially obtained from the *Banc de Sang i Teixits* (www.bancsang.net (accessed on 18 June 2022). Blood was not specifically collected for this research; the purchased units had been discarded for transfusion, usually because of an excess of blood relative to anticoagulant solution. Prior to their use, blood units underwent the analytical checks specified in the current legislation. Before being delivered to us, unit data were anonymized and irreversibly dissociated, and any identification tag or label was removed in order to guarantee the non-identification of the donor. No blood data were or will be supplied, in accordance with the current Spanish *Ley Orgánica de Protección de Datos* and *Ley de Investigación Biomédica*. The blood samples will not be used for studies other than those made explicit in this research. The studies reported here were performed under protocols reviewed and approved by the Ethics Committee on Clinical Research of the *Hospital Clínic de Barcelona* (Reg. HCB/2018/1223, 23 January 2019).

## 3. Results

### 3.1. Effect of DB on In Vitro P. falciparum Cultures

**Figure 3:**
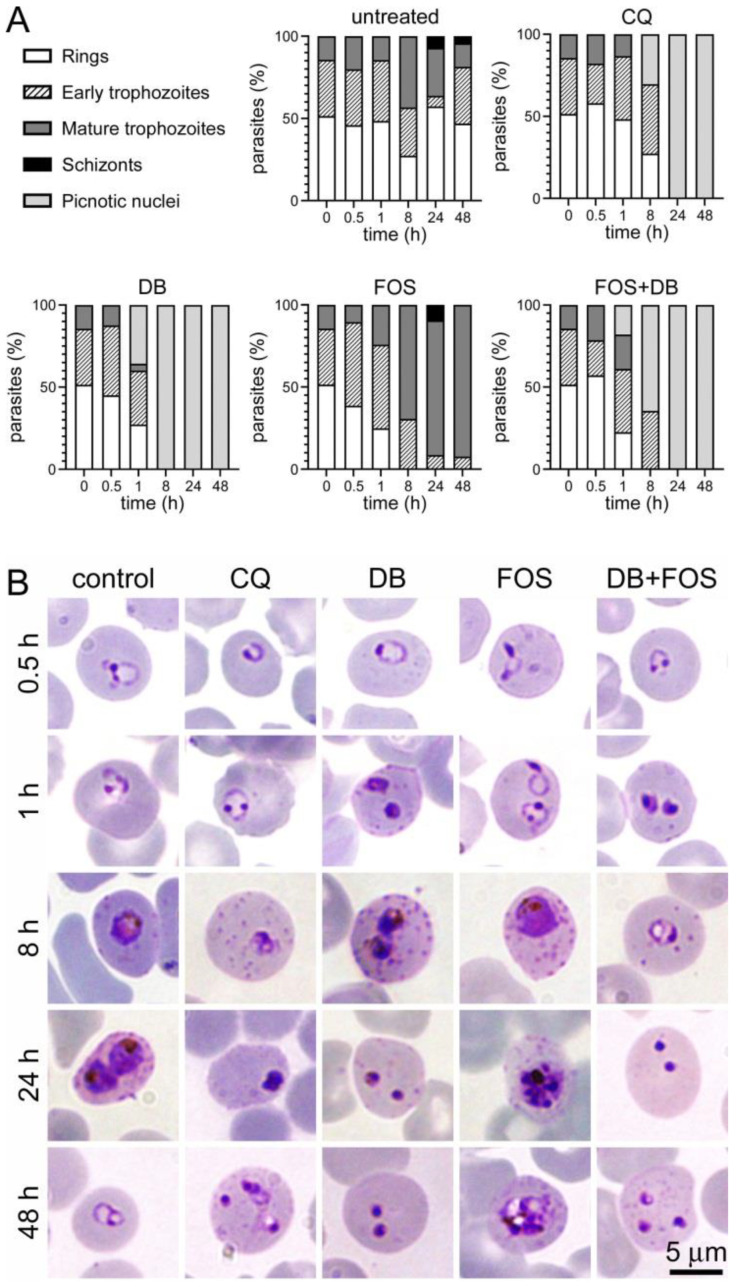
Effect of DB and fosmidomycin (FOS) on in vitro *P. falciparum* cultures. (**A**) Stage of growth inhibition of *P. falciparum* during 48 h of treatment with 20 µM DB, fosmidomycin, and both drugs combined. Controls included a non-treated sample and a culture treated with 20 µM chloroquine (CQ). Giemsa-stained blood smears were prepared at the indicated time points between 0 and 48 h of incubation, and the numbers of ring stages, early trophozoites, mature trophozoites, schizonts, and cells with picnotic nuclei were counted. Bars indicate the percentages of developmental stages present in the respective blood smears. (**B**) Representative images of Giemsa-stained *P. falciparum* blood stages at different times after the addition to the culture of antiplasmodial drugs.

The treatment of in vitro *P. falciparum* cultures with 20 µM DB and/or fosmidomycin, a concentration well above their IC50 values, resulted in different outcomes across time upon microscopic examination (Figure 3 and Figure S1). Whereas DB-treated samples 8 h after treatment showed clear growth inhibition of the parasite evidenced by the presence of abundant picnotic nuclei, cultures treated with fosmidomycin progressed until late trophozoite stages. DB + fosmidomycin-treated samples exhibited a pattern similar to that induced by DB alone, whereas chloroquine-treated controls presented the expected growth arrest at the early trophozoite stage.

### 3.2. Analysis of MEP Pathway and Citric Acid Cycle Metabolites in DB-Treated Samples

**Figure 4:**
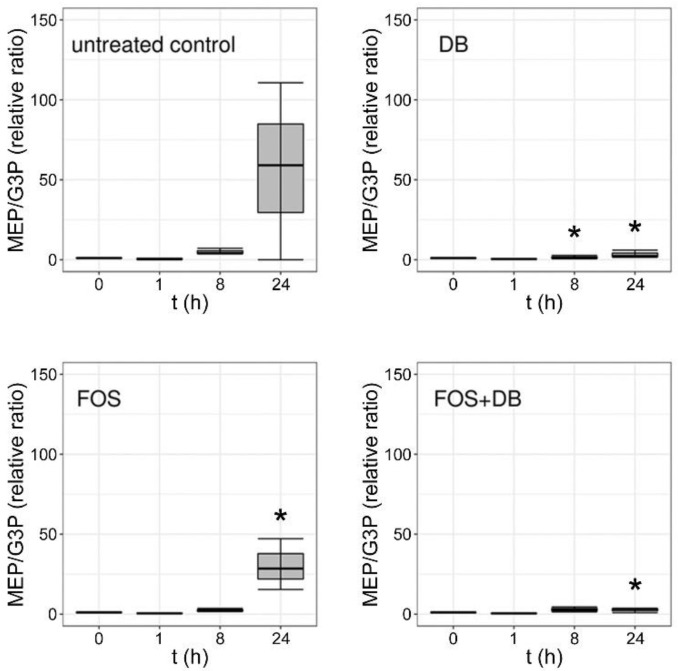
Relative MEP/G3P molar ratios in extracts at different times of *P. falciparum* cultures treated with 20 µM DB and fosmidomycin, individually and combined. *: *p* < 0.05.

**Figure 5:**
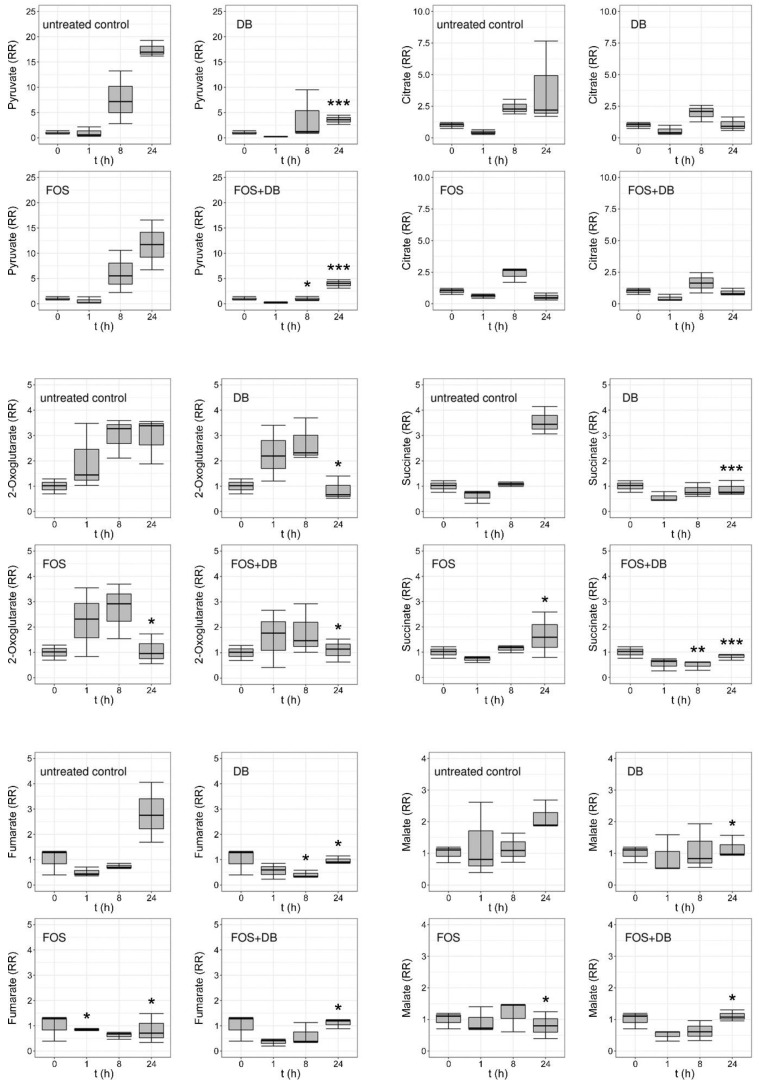
Relative amounts of pyruvate and citric acid cycle intermediates in extracts at different times of *P. falciparum* cultures treated with 20 µM DB and fosmidomycin, individually and combined. RR: relative response. *: *p* < 0.05; **: *p* < 0.005; ***: *p* < 0.0005.

**Figure 6:**
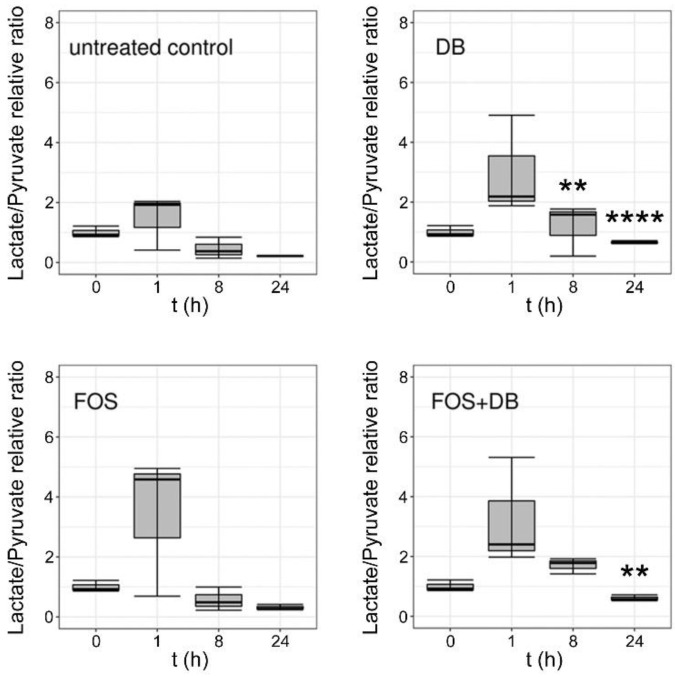
Relative lactate/pyruvate molar ratios in extracts at different times of *P. falciparum* cultures treated with 20 µM DB and fosmidomycin, individually and combined. **: *p* < 0.005; ****: *p* < 0.0001.

LC–ESI–MS/MS analysis of synchronized untreated *P. falciparum* control culture extracts revealed a significant rise in the MEP:G3P ratio 24 h after ring-stage synchronization (Figure 4), in agreement with the increased synthesis of MEP as the parasite grows and progresses through its intraerythrocytic cycle. Fosmidomycin led to a decrease in the MEP:G3P ratio relative to the control, as expected from the inhibition of DXR (see Figure 1) by this drug and the ensuing reduction in MEP levels. The effects of the DB treatment resulted in a complete arrest of MEP production, in agreement with the observed rapid parasite death upon administration of this drug to in vitro cultures. The DB-induced inactivation of CMS (see Figure 1) did not lead to the accumulation of MEP that would occur if the parasite had the rest of its metabolism intact, and maintained a regular supply of pyruvate and G3P entering the MEP pathway; this result was instead suggestive of widespread cellular damage in the pathogen. This fast-killing activity of DB was confirmed through analysis in treated cultures of the main metabolites of the citric acid cycle, for 24 h after synchronization at ring stages (Figure 5). Whereas in untreated cultures all the analyzed Krebs cycle intermediates increased, both fosmidomycin- and, especially, DB-treated cultures after 24 h exhibited low levels for citrate, 2-oxoglutarate, succinate, fumarate and malate. Upon treatment with DB, pyruvate was significantly reduced relative to the control, suggesting the activation of an alternative path for energy production following the observed citric acid cycle blockade. Indeed, analysis of the relative levels of lactate and pyruvate (Figure 6) indicated an increase, in comparison with the untreated culture, in lactate dehydrogenase activity in the presence of both drugs 1 h after treatment. Such an increment was significant for DB-containing samples at 8 h and, particularly, 24 h after treatment start, pointing to an activation of glycolysis in the dying parasites to compensate for the arrest of mitochondrial activity.

## 4. Discussion

The apicoplast organelle originated from the endosymbiotic association of *Plasmodium* evolutionary ancestors with a cyanobacterium [42]. This is the reason why antibiotics such as doxycycline, solithromycin, azithromycin, clindamycin, and chloramphenicol inhibit central apicoplastidic processes, such as genome replication, transcription, translation, and proteostasis [43,44]. Malaria parasites treated with these compounds do not die immediately, and are capable of producing invasive merozoites and therefore continuing with the asexual cycle of replication. However, the apicoplast of the next generation of parasites is not functional (if existent), because it has failed to replicate and segregate properly, which will make the parasite population collapse. This is known as a delayed-death phenotype and should be taken into account when screening for new antimalarial compounds [45]. In contrast, fosmidomycin and DB target the apicoplast metabolism, thus inhibiting the parasite development directly. This is of major importance, as in this case the therapeutic effect is obtained in the same cycle of replication where the drugs are administered, which results in a reduction in the pathogenic clinical effects of the parasite. In this work, we show that the effect of DB is already observed after 1 h of treatment, slowing down parasite growth in the early ring stage. This can translate into an added therapeutic advantage, as the parasitized erythrocytes containing picnotic rings might remain functional and could be pitted by the spleen, recovering them as functional cells [46]. Such a rapid parasite-killing profile could be interesting for combination therapy [47], which the World Health Organization recommends as the optimal drug administration strategy in uncomplicated malaria [48]. In this approach, two or more antimalarials [49] with different antiparasitic mechanisms are combined, one with a fast-killing action (usually artemisinin or some of its derivatives, with elimination half-lives of approximately 1–3 h [50]) and a second compound with a longer blood circulation time (between 4 days and several weeks [51]) to finish off the surviving parasites. However, the incipient resistance to artemisinin detected in several malaria-endemic regions demands new rapidly acting drugs, for which DB could be a good candidate. The mode of action of DB is the inhibition of CMS, leading to rapid parasite death through a subsequent disruption of the citric acid cycle. However, working out the details of the precise molecular mechanisms will require a more extensive analysis through dose-response metabolomics studies at different DB concentrations throughout an expanded time frame.

In malaria parasites, IPP and DMAPP are building blocks used to synthesize small-molecule isoprenoids with a host of functions, or C15/C20 prenyl chains for the post-translational modification of proteins [52,53]. Essential isoprenoid products in *Plasmodium* include ubiquinone, a component of the mitochondrial electron transport chain [54], dolichols involved in protein N-glycosylation [55], and vitamin E, which is one of the key parasite defenses against oxidative stress induced by pro-oxidant compounds such as artemisinin [56]. Nevertheless, recent studies indicate that the key essential function of isoprenoids in the parasite blood stages is their role as a substrate for protein prenylation, an important post-translational modification that regulates protein targeting and function throughout the cell [53,57,58]. Whereas most studied organisms make wide use of protein prenylation, malaria parasites have a small prenylated blood stage proteome, consisting primarily of proteins driving vesicular transport to the digestive vacuole [53,57], notably the Rab family GTPases [59,60]. In the absence of prenylation, Rab5 trafficking is disrupted, which leads to digestive vacuole destabilization and parasite death [57].

Among the heat shock proteins (HSP) that are necessary for protein folding and stabilization, the robust prenylation of HSP40 during intraerythrocytic replication was found to be critical for *P. falciparum*’s survival of thermal stress [58]. The inhibition of isoprenoid biosynthesis resulted in the reduced association of HSP40 with critical components of the cytoskeleton, protein export, and vesicular transport pathways, without which *P. falciparum* could survive neither heat nor cold stress. Other reports also showed that apicoplastidic isoprenoid biosynthesis is one of the essential metabolic pathways involved in the parasite survival response to the extreme conditions of the host’s malarial fever [61].

DB is a potent inhibitor of human ether-a-go-go-related gene (HERG) potassium channels [62], and it also affects the activity of some hydrolytic enzymes [63]. These potential side effects call for delivery strategies based on the encapsulation of this drug in nanocarriers targeted to *Plasmodium*-infected cells, which will allow for high local parasite-killing concentrations while maintaining the overall administered dose below the lowest published lethal dose (10 mg/kg) [30,31]. DB has a log *P* of 2.55 (https://chemaxon.com (accessed on 18 June 2022), indicating a high lipophilicity and therefore a wide biodistribution, although this characteristic of the drug might also offer a potential solution to this limitation. The long hydrocarbon tail and charged head of DB are molecular features that resemble those of membrane lipids, thus suggesting that this compound could be efficiently incorporated into liposomes to improve its delivery [28]. Indeed, targeted delivery will likely be essential for drugs such as DB which must reach the apicoplast, because, in addition to the organelle’s membrane, three other lipid bilayers must be crossed, namely those of the parasitized red blood cell, the parasitophorous vacuole containing the parasite, and the plasma membrane of *Plasmodium*. A targeted delivery strategy will also contribute to reduce the relatively high IC50 of this drug for *Plasmodium* (1 µM) [28]. Previous data showed that DB had a disruptive effect on liposomal lipid bilayers at in vitro concentrations close to those required for its antiplasmodial activity in *P. falciparum* cultures [28]. This indicates that DB encapsulation in nanocarriers will require either the adaptation of the lipid formulation of liposomes to impart upon their membranes a higher stability, or the use of non-liposomal drug carriers, such as the different types of polymeric nanoparticles that have shown efficiency in the targeted delivery of antiplasmodial drugs [38,64,65]. The next steps in the eventual pharmaceutical development of such DB nanoformulations will need to include pharmacokinetics/pharmacodynamics analyses and in vivo assays in murine models of malaria.

## 5. Conclusions

The results reported above present DB as a fast-killing antiplasmodial whose effects are already felt by the parasite 1 h after treatment start. The MEP pathway blockade by DB is mirrored by a simultaneous arrest of the citric acid cycle and siphoning of pyruvate towards glycolysis. The relatively high in vitro IC50 of this drug could be improved through targeted delivery nanoencapsulation strategies to facilitate its entry into parasitized erythrocytes and towards its target enzyme inside the apicoplast organelle. If this current limitation can be solved, DB might become an important actor in future antimalarial combination therapies.

## Data Availability

The data presented in this study are available on request from the corresponding author.

## Supplementary Materials

The following are available online at https://www.mdpi.com/article/10.3390/pharmaceutics14071320/s1, Figure S1: Effect of DB and fosmidomycin (FOS) on in vitro *P. falciparum* cultures.
