# Supplementary material for: Characterization of Domiphen Bromide as a New Fast-Acting Antiplasmodial Agent Inhibiting the Apicoplastidic Methyl Erythritol Phosphate Pathway
Source: Pharmaceutics. 2022 Jun 22;14(7):1320. doi: 10.3390/pharmaceutics14071320 (PMC9319574; doi:10.3390/pharmaceutics14071320)
Supplement: Supplementary file 1 [file pharmaceutics-14-01320-s001.zip › pharmaceutics-1751244-supplementary.pdf]

Characterization of Domiphen Bromide as a New Fast-acting Antiplasmodial Agent Inhibiting the Apicoplastidic Methyl Erythritol Phosphate Pathway

Arnau Biosca [1,2,3,#], Miriam Ramírez [1,#], Alex Gomez-Gomez [4,5], Aritz Lafuente [1,2,3], Valentín Iglesias [1,2,3,6], Oscar J. Pozo [4,5], Santiago Imperial [3,7], Xavier Fernàndez-Busquets [1,2,3,*]

[1] Barcelona Institute for Global Health (ISGlobal, Hospital Clínic-Universitat de Barcelona), Rosselló 149-153, 08036 Barcelona, Spain; naubiosca@gmail.com (A.B.), miriam.ramirez@isglobal.org (M.R.), aritz.lafuente@gmail.com (A.L.), valentin.iglesias.mas@gmail.com (V.I.)
[2] Nanomalaria Group, Institute for Bioengineering of Catalonia (IBEC), The Barcelona Institute of Science and Technology, Baldiri Reixac 10-12, 08028 Barcelona, Spain
[3] Nanoscience and Nanotechnology Institute (IN2UB), University of Barcelona, Martí i Franquès 1, 08028 Barcelona, Spain; simperial@ub.edu (S.I.)
[4] Integrative Pharmacology and Systems Neuroscience Research Group, Neurosciences Research Program, IMIM-Institut Hospital del Mar d'Investigacions Mèdiques, Doctor Aiguader 88, 08003 Barcelona, Spain; agomez@imim.es (A.G.-G.), opozo@imim.es (O.J.P.)
[5] Department of Experimental and Health Sciences, Universitat Pompeu Fabra, Doctor Aiguader 88, 08003 Barcelona, Spain
[6] Institut de Biotecnologia i Biomedicina and Departament de Bioquímica i Biologia Molecular, Universitat Autònoma de Barcelona, 08193 Bellaterra, Spain
[7] Department of Biochemistry and Molecular Biomedicine, University of Barcelona, Avda. Diagonal 643, 08028 Barcelona, Spain

# Both authors contributed equally
* Correspondence: xfernandez_busquets@ub.edu (X.F.-B.); Tel.: +34-93-227-5400 (extension 4581)

Supplementary Material

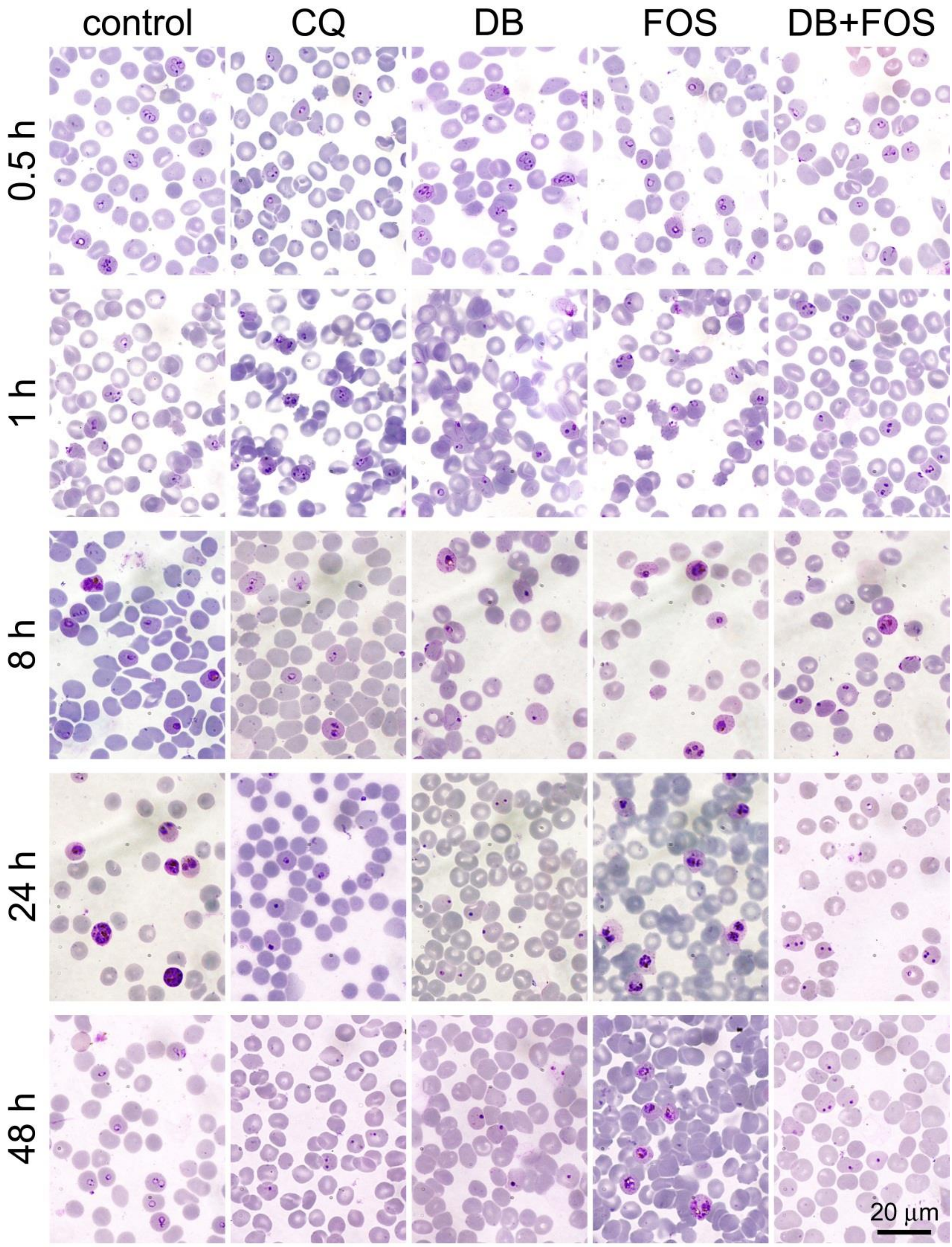

Figure S1. Effect of DB and fosmidomycin (FOS) on *in vitro P. falciparum* cultures. Representative images of Giemsa-stained *P. falciparum* cultures at different times after the addition of antimalarial drugs. CQ: chloroquine.
